# *Schistosoma japonicum* SjE16.7 Protein Promotes Tumor Development via the Receptor for Advanced Glycation End Products (RAGE)

**DOI:** 10.3389/fimmu.2020.01767

**Published:** 2020-08-21

**Authors:** Chenyun Wu, Xinyue Du, Lili Tang, Jianhua Wu, Wei Zhao, Xiaokui Guo, Dengyu Liu, Wei Hu, Helena Helmby, Guangjie Chen, Zhaojun Wang

**Affiliations:** ^1^Department of Immunology and Microbiology, Shanghai Jiao Tong University School of Medicine, Shanghai, China; ^2^School of Global Health, Chinese Center for Tropical Diseases Research, Shanghai Jiao Tong University School of Medicine, Shanghai, China; ^3^Department of Basic Medicine, Guangxi Medical University, Nanning, China; ^4^School of Life Sciences, Fudan University, Shanghai, China; ^5^Department for Infection Biology, London School of Hygiene and Tropical Medicine, London, United Kingdom

**Keywords:** schistosome, calcium binding protein, colorectal cancer, RAGE, inflammation

## Abstract

Schistosome infection contributes to cancer development, but the mechanisms are still not well-understood. SjE16.7 is an EF-hand calcium-binding protein secreted from *Schistosoma japonicum* eggs. It is a neutrophil attractant and macrophage activator and, as such, plays an important role in the inflammatory granuloma response in schistosomiasis. Here, we show that SjE16.7 binds to host cells by interacting with receptors for advanced glycation end products (RAGE). This ligation leads to activation of the NF-κB signaling pathway, an increase in the generation of reactive oxygen species, and production of the pro-inflammatory cytokines IL-6 and TNF-α. Using a mouse model of colorectal cancer, we demonstrate that intraperitoneal injection of SjE16.7 promotes colorectal cancer progression along with systemic myeloid cell accumulation. Thus, our results identify a new helminth antigen contributing to tumor development in the mammalian host.

## Introduction

More than 15% of cancers in the world are caused by infectious agents (1, 2). Aside from viruses, bacteria, and fungi, parasites are also important biological carcinogens. Schistosomiasis, caused by infection with blood fluke trematodes of the genus *Schistosoma*, is one of the most important human parasitic diseases across the world. More than 200 million cases of schistosomiasis are currently estimated worldwide (3). Schistosomiasis is associated with development of urinary bladder cancer, colorectal cancer, rectal cancer, and hepatocellular carcinoma (4). *Schistosoma haematobium* has been classified as a definite carcinogen to human (group I carcinogen), and *S. japonicum* is considered a possible carcinogen for human (group 2B carcinogen) (5). It is generally accepted that chronic inflammatory processes and oxidative stress are responsible for the role of schistosome infection in carcinogenesis (4). However, the identity of the carcinogenic parasite-derived molecules as well as the underlying mechanisms responsible for tumor development are still not well-understood.

SjE16.7 is an EF-hand calcium-binding protein derived from the eggs produced by *S. japonicum*. We have previously demonstrated that SjE16.7 is recognized by the host immune system, attracting neutrophils and initiating the inflammatory response seen in schistosomiasis (6). SjE16.7 also promotes chronic inflammation by inducing macrophage chemotaxis and cytokine production, and it is involved in the development of the schistosome egg granuloma (7). In mammalian cells, EF-hand proteins, such as S100 proteins, are highly associated with inflammation and tumorigenesis (8–10). They bind to receptors for advanced glycation end products (RAGE), a multiligand receptor of the immunoglobin family, and are involved in the regulation of leukocyte trafficking and inflammation (11). RAGE ligation results in the activation of a diverse range of signal transduction cascades and downstream pathways, including NF-κB, and the generation of ROS and acts as a key player in bridging inflammatory processes and cancer development (11–13). In this study, we show that the binding of SjE16.7 to RAGE plays a critical role in inducing activation of myeloid inflammatory cells. Furthermore, SjE16.7 promotes chronic myeloid cell accumulation and colitis-associated colorectal cancer development in an *in vivo* mouse model. Thus, our results identify a new helminth antigen contributing to tumor development in the mammalian host.

## Materials and Methods

### Ethics Statement

The conducts and procedures involving animal experiments were approved by the Animal Ethics Committee of Shanghai Jiao Tong University School of Medicine (project number A-2016-028) according to Regulations for the Administration of Affairs Concerning Experimental Animals (approved by the State Council of the People's Republic of China) and Guide for the Care and Use of Laboratory Animals (Department of Laboratory Science, Shanghai Jiao Tong University School of Medicine, laboratory animal usage license number SYXK 2013-0050, certified by Shanghai Committee of Science and Technology).

### Mice

C57BL/6 mice (male, 6–8 weeks old) were purchased from Shanghai Laboratory Animal Center, Chinese Academy of Sciences. Mice were housed in the Shanghai Jiao Tong University School of Medicine Animal Care Facilities under specific pathogen-free conditions.

### Materials

Chemicals were purchased from Sigma-Aldrich Co. unless otherwise noted. Ca^2+^, phosphate-buffered saline (PBS; pH 7.2), Dulbecco's Modified Eagle Medium (DMEM), fetal bovine serum (FBS), penicillin/streptomycin, L-glutamine, and EDTA were obtained from Life Technologies. Glutathione sepharose 4B was ordered from GE healthcare, Stockholm, Sweden. Antibodies used in Western blot analysis included goat anti-mouse RAGE antibody (R&D systems, Apolis, MN, USA), mouse anti-GAPDH antibody (Sigma, Saint Louis, MO, USA), rabbit anti-β-actin antibody (Cell Signaling Technology, Boston, MA, USA), rabbit anti-GST antibody (Millipore, Bedford, MA, USA), rabbit anti-RAGE1, anti-phosphor-IκB, Phospho-p65, total IκB and p65 antibody (Cell Signaling Technology, Boston, MA, USA), and HPR-conjugated anti-mouse or anti-rabbit IgG (Cell Signaling Technology, Boston, MA, USA). FITC-conjugated anti-mouse B220, APC-conjugated anti-mouse CD3, FITC or PerCP-conjugated anti-mouse CD4, PE-conjugated anti-mouse CD8, cFITC-conjugated anti-mouse CD11b, PerCP-conjugated anti-mouse Gr-1 (Ly6C and Ly6G), PE-conjugated anti-mouse Foxp3, FITC-conjugated anti-mouse IFN-γ, PE-conjugated anti-mouse IL-4, PerCP-conjugated anti-mouse IL-17 and control isotype antibodies used for flow cytometry were ordered from eBioscience (San Diego, CA, USA).

Recombinant antigens (His-SjE16.7 from yeast expression system, GST, GST-SjE16.7 from *E. coli*), GST, and GST-SjE16.7-conjugated glutathione sepharose 4B beads were prepared by our own lab as described previously.

### Cell Culture

The murine RAW 264.7, human Caco-2, and SW480 (ATCC) cell lines were cultured in Dulbecco's modified Eagle's medium (DMEM) containing 10% heat-inactivated FBS, 2 mM L-glutamine, and 100 U/ml penicillin/streptomycin (D10).

To generate bone marrow–derived macrophages (BMDMs), bone marrow cells were harvested from mice and cultured in D10 with 50 ng/ml M-CSF for 7 days. The resting macrophages were collected using 5 mM EDTA in cold PBS. After centrifugation, the cells were resuspended in D10, seeded on a plate, and rested for 24 h before functional assays. BMDMs were > 95% CD11b^+^ and F4/80^+^ as determined by flow cytometry.

### Conjugation of Proteins to FITC and Staining of Cells

Purified proteins were conjugated to FITC using the FluoroTag FITC conjugation kit (Sigma) as per the manufacturer's protocol. Incubation of FITC-tagged proteins and cells were done in the presence of 1% non-fat dry milk in PBS for 20 min at 4°C, followed by repeated washings before analysis by flow cytometry.

### Flow Cytometry

For surface staining, cell suspensions (1 × 10^6^ cells) were washed once in FACS buffer (0.5% BSA-PBS), incubated with antibodies for 30 min at 4°C, followed by two washes with FACS buffer and fixation using 0.5% paraformaldehyde/FACS buffer. For intracellular staining, after the standard steps, cell suspensions were washed twice in PBS containing 0.5% saponin (Sigma), then incubated with antibodies in 0.3% saponin for 30 min at 4°C, followed by two washes with FACS buffer and fixation using 0.5% paraformaldehyde/FACS buffer. Foxp3 staining was performed using Foxp3/Transcription Factor Staining Buffer Set according to the manufacturer's instructions (eBioscience, San Diego, CA, USA). All antibodies were used at 1 μg/ml. Flow cytometric data was acquired on a BD FACS Calibur Flow Cytometer and analyzed using FlowJo software.

### GST-Pulldown

RAW 264.7, Caco-2, and SW480 cell lysates were prepared using lysis buffer (50 mM HEPEs, 150 mM NaCl, 1 mM EDTA, pH 7.0, 0.1% IGEPAL) supplemented with 1× complete protease inhibitors mixture (Roche). Protein concentration was determined by BCA assay (Pierce). GST pulldown was performed using GST and GST-SjE16.7 sepharose beads. The cell lysates were incubated with 30 μl GST or GST-SjE16.7 beads for 1 h at 4°C, followed by washing of the beads. Total cell lysates and bead-bound proteins were subjected to Western blot.

### Western Blot Analysis

Proteins were separated by 10–12% SDS-PAGE. Western blotting was performed on nitrocellulose filter (Biorad). The membranes were blocked for 1 h in TBST (10 mM Tris pH 8.0, 150 mM NaCl, 0.1% Tween 20) with 5% non-fat dry milk and incubated with primary antibodies overnight, followed by washing and incubation with HRP-conjugated secondary antibodies. Enhanced chemiluminescence (ECL, Pierce) was used as substrate, and the signals were analyzed by Luminescent imager (ImageQuant Las 4000, GE Healthcare).

### shRNA Knockdown of RAGE Expression

The constructs in pGMLV-SC5, containing RAGE-specific short hairpin RNA (shRNA) sequences directed to mouse RAGE mRNA (GeneBANK accession no. NM_007425) and non-specific shRNA control vector were purchased from Genomeditech Co. Ltd. (Shanghai, China). RAW 264.7 cells were transfected using Attractene transfection reagent according to the manufacturer's instruction (QIAGEN). Cells were used for the experiments 24–48 h after transfection, and the RAGE expression level following shRNA knockdown was assessed by real-time PCR and Western blot.

### Detection of Reactive Oxygen Species (ROS)

ROS production by BMDMs was measured by a luminol-dependent chemiluminescence assay, and 3 × 10^5^ cells were plated in a 96-well luminometer plate (Costar) and prewarmed for 5 min. Prewarmed His-SjE16.7, GST, or GST-SjE16.7 (1 μM) were added with luminol (100 μM) and HRP (20 U/ml) at the same time, and measurements started immediately. Chemiluminescence was measured at 2.5-min intervals for 30–60 min with a luminometer (BioTek Synergy HT microplate reader).

The ROS production of RAW 264.7 cells was detected using 2′,7′-dichlorofluorescein diacetate (DCFDA). Cells were incubated with the fluorogenic probe DCFDA for 30 min at 37°C in 5% CO_2_. ROS levels were determined using a microplate reader.

### Real-Time Quantitative PCR

BMDMs or RAW 264.7 cells were stimulated with 1 μM recombinant His-SjE16.7, GST-SjE16.7, or control recombinant protein GST in D10 for 2 h. Total RNA was isolated using RNeasy Kits (Roche), and first strand cDNA was subsequently synthesized with random hexamer oligonucleotides using Invitrogen reverse transcriptase II. Real-time quantitative PCR was carried out in an Applied Biosystems 7500 system using Power SYBR Green PCR Master Mix (Applied Biosystems). Thermocycler conditions comprised an initial holding at 50°C for 2 min and a subsequent holding at 95°C for 10 min, which was followed by a two-step PCR program at 95°C for 15 s and 60°C for 60 s for 40 cycles. Relative levels of gene expression of IL-1β, IL-6, and TNF-α were determined using GAPDH as the control. Sequences of the PCR primer pairs were as follows:

GAPDH forward 5′-CTGAGCAAGAGAGGCCCTATCC-3′;

GAPDH reverse 5′-CTCCCTAGGCCCCTCCTGTT-3′;

IL-6 forward 5′- TAGTCAATTCCAGAAACCGCTATG -3′;

IL-6 reverse 5′- GTAGGGAAGGCCGTGGTTGT -3′;

TNF-α forward 5′- GACGTGGAACTGGCAGAAGAG -3′;

TNF-α reverse 5′- GCCACAAGCAGGAATGAGAAG -3′.

### Induction of Colitis Associated Colon Cancer (CAC) in Mice

The colitis-associated colon cancer model was induced with dextran sodium sulfate (DSS) (36–50 kDa; MP Biomedicals, CA, USA) and azoxymethane (AOM) (Sigma). Mice were injected intraperitoneally (i.p.) with a single dose of 10 mg/kg AOM on day 1. After the AOM injection, mice were given two cycles of 4% DSS (cycle 1: days 1–7; cycle 2: days 15–21) in the drinking water, followed by normal drinking water until the end of the experiment. Recombinant proteins (100 μg His-SjE16.7, GST-SjE16.7, or GST) were injected i.p. twice a week for the duration of the experiment.

### Enzyme-Linked Immunosorbent Assay (ELISA)

Supernatants were collected for measurement of TNF-α and IL-6, using ELISA kits (R&D Systems) according to the manufacturer's instructions. A standard curve was generated using known amounts of the respective purified recombinant murine cytokine.

### Statistical Analysis

Flow cytometry, chemiluminescence, 2′7′-dichoroluorescein (DCFH or DCF), real-time PCR, and tumor number data were analyzed by unpaired Student's *t*-test using GraphPad Prism software. Data are typically expressed as the mean ± SEM. Significance parameters were set at *p* < 0.05.

## Results

### RAGE Is the Receptor for SjE16.7 on Host Cells

**Figure 1:**
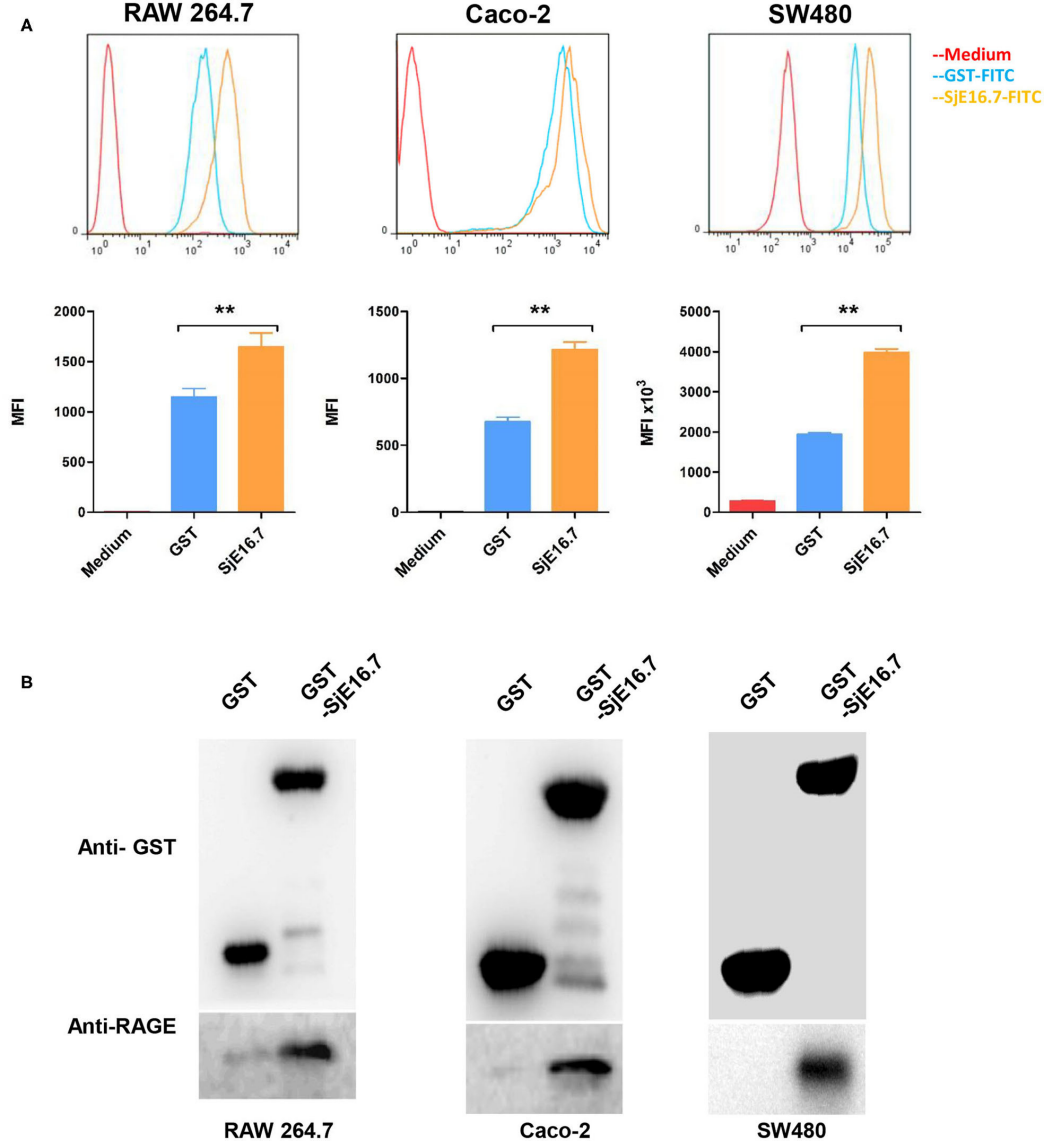
SjE16.7 binds to RAGE. **(A)** FITC-labeled SjE16.7 or control FITC-GST protein binding to RAW 264.7, Caco-2, and SW-480 cells was measured by flow cytometry. Pooled data from two independent experiments are shown. MFI, Mean Fluorescence Intensity. **p < 0.01 FITC-SjE16.7 group compared with FITC-GST group. **(B)** RAW 264.7, Caco-2, and SW-480 cell lysates were prepared and pulled down with GST or GST-SjE16.7 sepharose beads. Protein on beads and in total cell lysates was subjected to Western blot to determine the levels of bound RAGE. The experiments were repeated at least three times with similar results.

To investigate the mechanism by which SjE16.7 triggers host cell activation, we analyzed whether SjE16.7 can bind to host cells. FITC-labeled recombinant SjE16.7 or control GST proteins were incubated with different host cells, and after removal of unbound protein by extensive washing, the cells were analyzed by flow cytometry. As shown in Figure 1A, SjE16.7 showed robust binding to mouse macrophage cells (RAW 264.7), and the MFI of SjE16.7-labeled cells was significantly higher than that of control GST protein-labeled cells. Similar results were obtained using colorectal adenocarcinoma cells (Caco-2 and SW480), demonstrating that SjE16.7 binds to mammalian host cells more efficiently than control GST protein.

We then asked whether SjE16.7 can interact with RAGE as other EF-hand proteins, and for this purpose, we used a GST pulldown assay. Recombinant GST or GST-SjE16.7 proteins were incubated with cell lysates from RAW 264.7, Caco-2, or SW480 cells. After washing of the beads, the bound proteins were subjected to Western blot analysis. As shown in Figure 1B, SjE16.7 interacted with RAGE from mammalian cells.

### SjE16.7 Promotes ROS Production via RAGE

**Figure 2:**
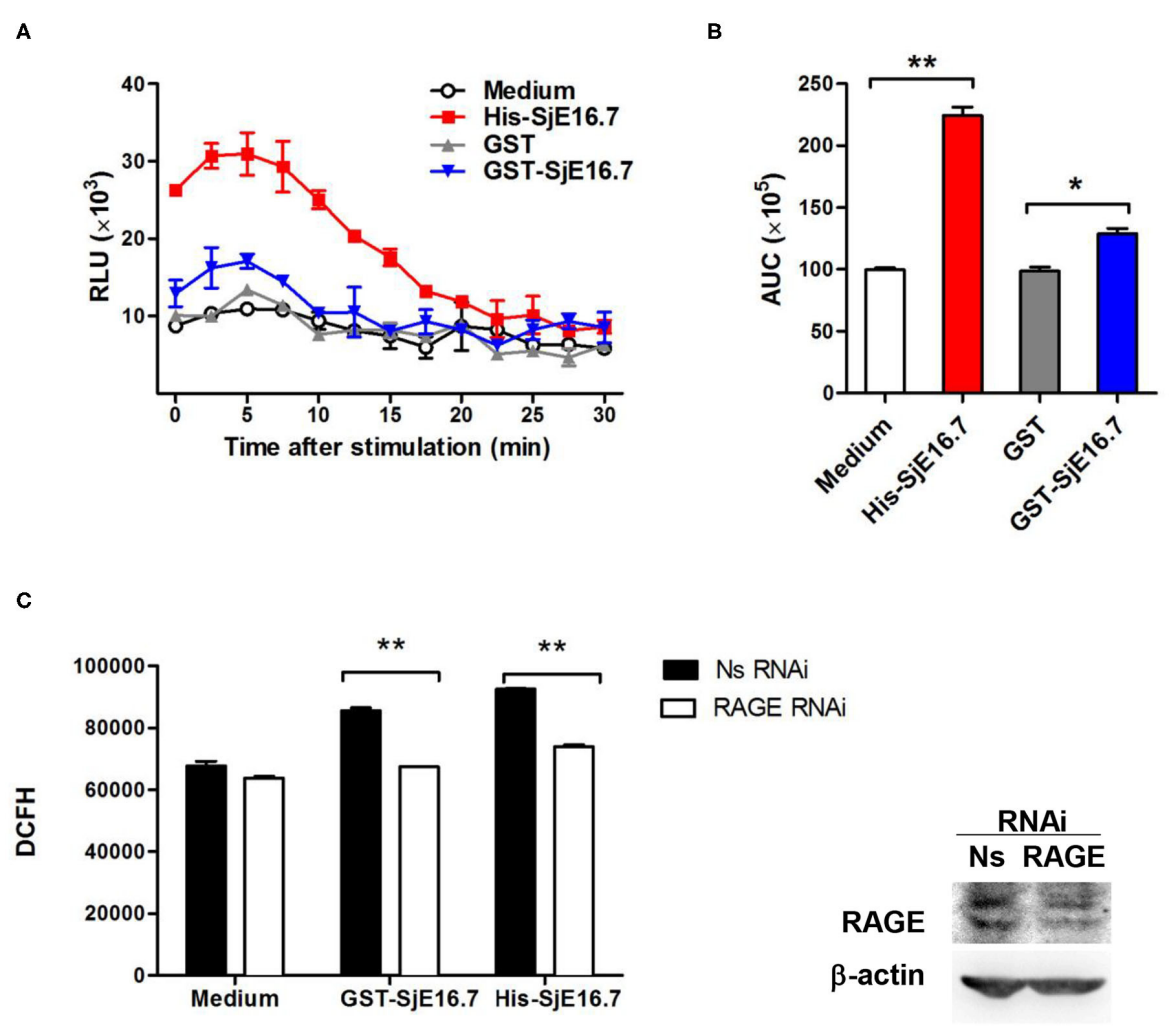
SjE16.7 promotes ROS production via RAGE. **(A)** BMDMs were stimulated with 1 μM eukaryotic recombinant protein His-SjE16.7 or prokaryotic recombinant protein GST-SjE16.7 GST, or GST control. ROS production was measured by chemiluminescence assay as described in section Materials and Methods. Data shown are profiles of the kinetics of ROS production. RLU, relative light unit. **(B)** Area under curve (AUC) in **(A)** is shown. **(C)** The expression of RAGE in RAW 264.7 cells was knocked down by lentivirus-mediated RNA interference (RNAi), and then the effect of SjE16.7 on ROS production was determined by DCFH assay (left panel). RAGE expression levels were measured by Western blot (right panel). Statistical analysis was performed on pooled data from three independent experiments. *p < 0.05, **p < 0.01 compared with indicated control group.

In order to investigate if the interaction between SjE16.7 and RAGE resulted in ROS production, we measured ROS production in SjE16.7 treated cells. Using a horseradish peroxidase (HRP)-dependent chemiluminescence assay, we found that both eukaryotic recombinant His-SjE16.7 and prokaryotic recombinant GST-SjE16.7 significantly increased ROS production in bone marrow–derived macrophages (BMDMs) compared to medium control or GST control protein (Figures 2A,B). To further confirm the importance of RAGE in SjE16.7-induced ROS production, we utilized a RAGE knockdown. The expression of RAGE in RAW 264.7 cells was blocked by lentivirus-mediated RNA interference (RNAi), and the effects of SjE16.7 on ROS production was determined by DCFH assay. The results shown in Figure 2C demonstrate that treatment of RAW 264.7 cells with SjE16.7 increased ROS production; however, when using RAGE knockdown cells, ROS production was significantly reduced back to background levels. Taken together, our results demonstrate that the interaction of SjE16.7 and RAGE results in increased ROS production by myeloid cells.

### SjE16.7 Induces NF-κB Signaling Activation and Proinflammatory Cytokine Production via RAGE

**Figure 3:**
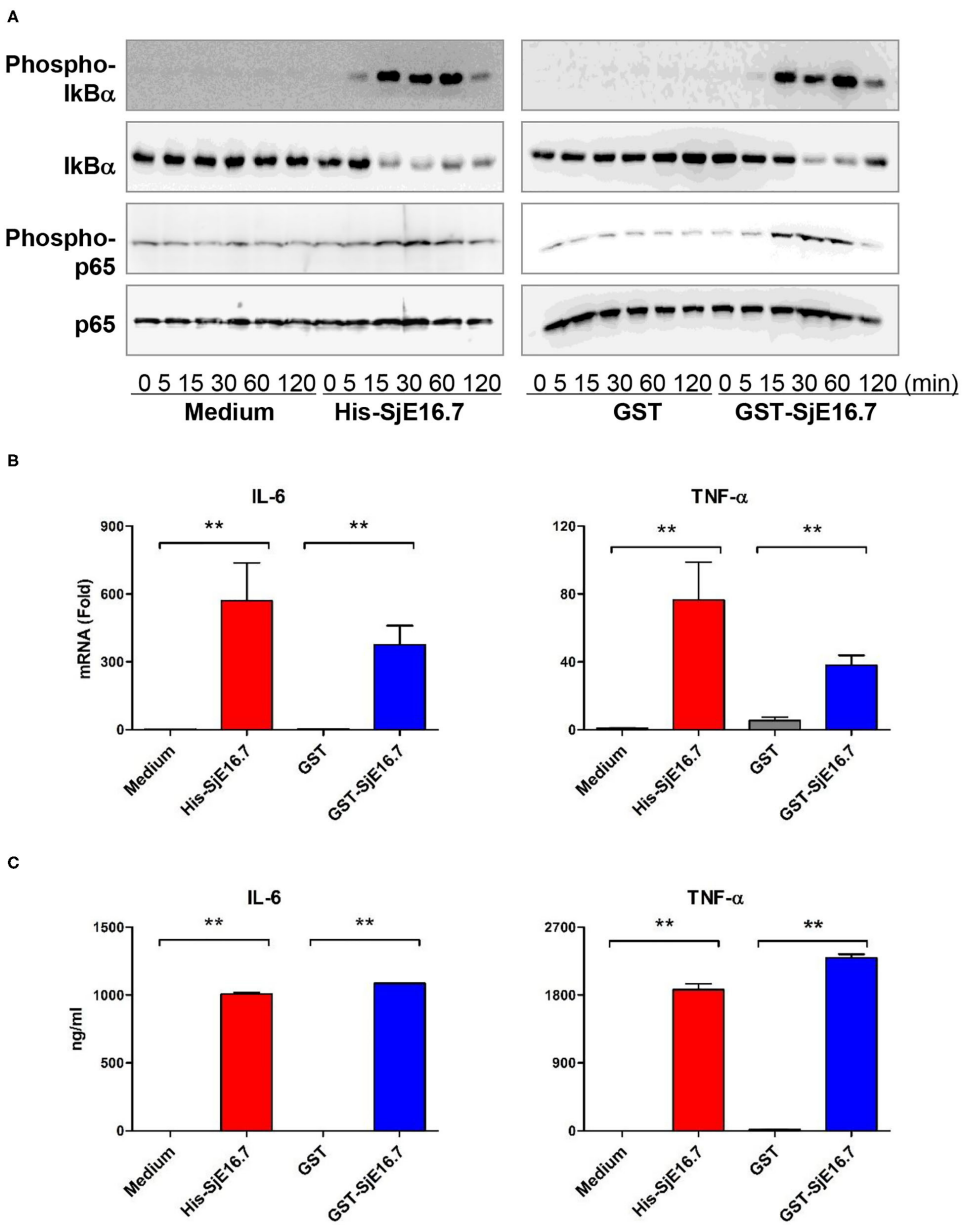
SjE16.7 induces NF-κB signaling activation and proinflammatory cytokine production. **(A)** BMDMs were stimulated with 1 μM eukaryotic recombinant protein His-SjE16.7 or prokaryotic recombinant protein GST-SjE16.7, or GST control, for the indicated times, and the levels of phospho-IκBα, total IκBα, phospo-p65, and total p65 were determined by Western blot analysis. Results are representative of three experiments performed. **(B)** BMDMs were stimulated with 1 μM recombinant His-SjE16.7, GST-SjE16.7, or control GST protein for 2 h, and IL-6 and TNF-α mRNA levels were determined by real-time PCR. **(C)** BMDMs were stimulated with 1 μM recombinant His-SjE16.7, GST-SjE16.7, or control GST protein for 24 h, and IL-6 and TNF-α protein levels in culture supernatant were determined by ELISA. Statistical analysis was performed on pooled data from two to four independent experiments. **p < 0.01.

**Figure 4:**
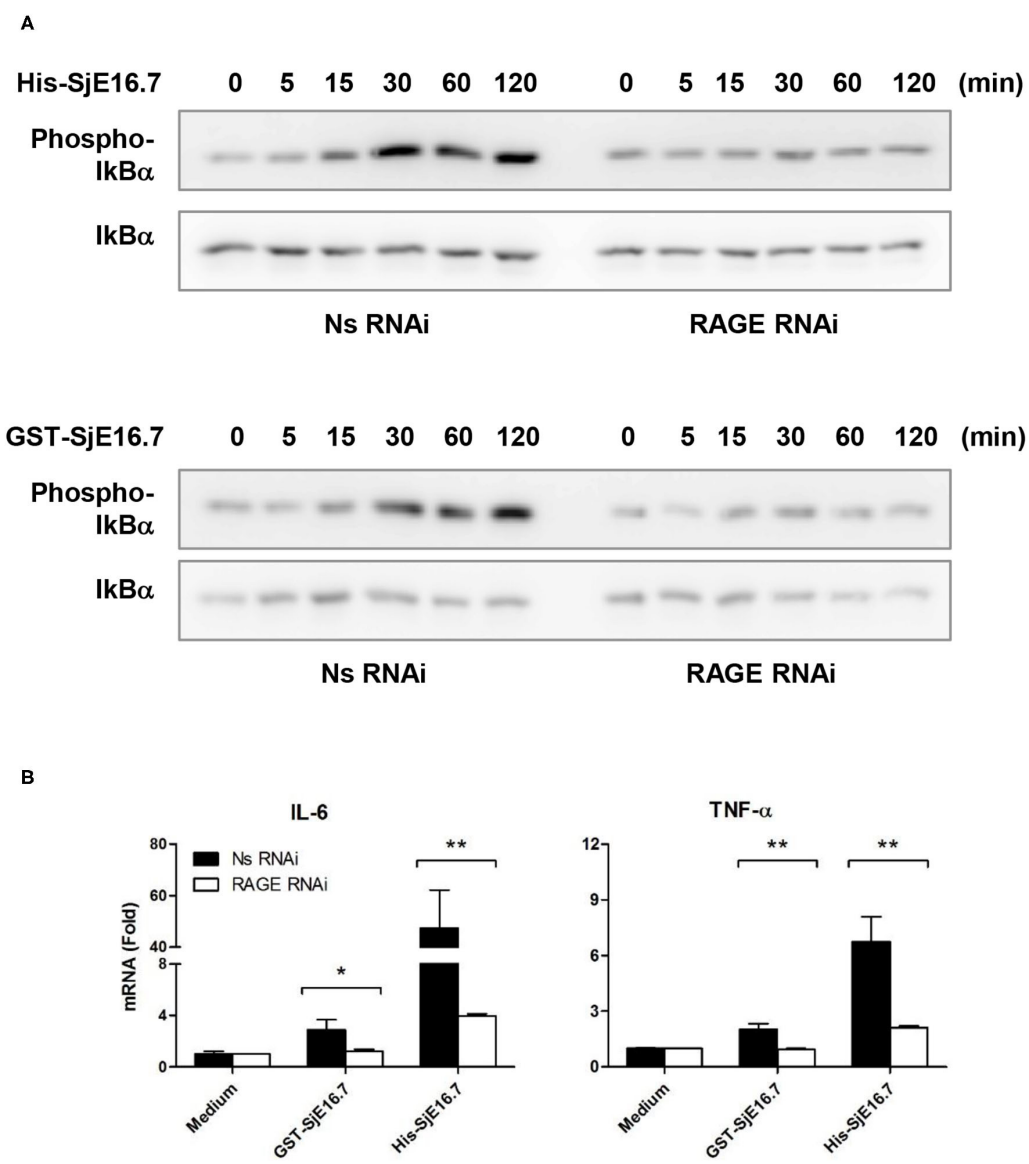
RAGE is essential for SjE16.7-induced NF-κB signaling activation and proinflammatory cytokine production. **(A)** The expression of RAGE in RAW 264.7 cells was knocked down by lentivirus-mediated RNA interference (RNAi). The cells were then stimulated with 1 μM eukaryotic recombinant protein His-SjE16.7 or prokaryotic recombinant protein GST-SjE16.7 for the indicated times. The levels of phospho-IκBα and total IκBα, phospo-p65 and total p65 were determined by Western blot. Results are representative of two experiments. **(B)** RAGE knockdown RAW 264.7 cells or control cells were stimulated with 1 μM recombinant His-SjE16.7 or GST-SjE16.7 for 2 h. IL-6 and TNF-α mRNA levels were determined by real-time PCR. Statistical analysis was performed on pooled data from three independent experiments. *p < 0.05, **p < 0.01.

We next analyzed the effects of SjE16.7 on IκB and NF-κB activation in mouse BMDMs. Phosphorylated and total IκBα were determined by Western blot analysis. As shown in Figure 3, eukaryotic His-SjE16.7 and prokaryotic GST-SjE16.7 induced a rapid phosphorylation of IκB and degradation of total IκB protein. Moreover, both eukaryotic His-SjE16.7 and prokaryotic GST-SjE16.7 induced more phosphorylated p65 subunit of NF-κB than medium control or GST control protein (Figure 3A). The stimulation of BMDMs with SjE16.7 led to a significant increase in proinflammatory cytokine expression (IL-6 and TNF-α) at both mRNA and protein levels (Figures 3B,C). However, when using RAGE knockdown RAW 264.7 cells, there was less NF-κB signaling activation and proinflammatory cytokine production compared to the control cells (Figure 4), suggesting that NF-κB activation and the resulting pro-inflammatory cytokine release induced by SjE16.7 was mediated through its interaction with RAGE.

### SjE16.7 Promotes Increased Incidence of Colitis-Associated Colorectal Cancer and Splenic Myeloid Cell Accumulation

**Figure 5:**
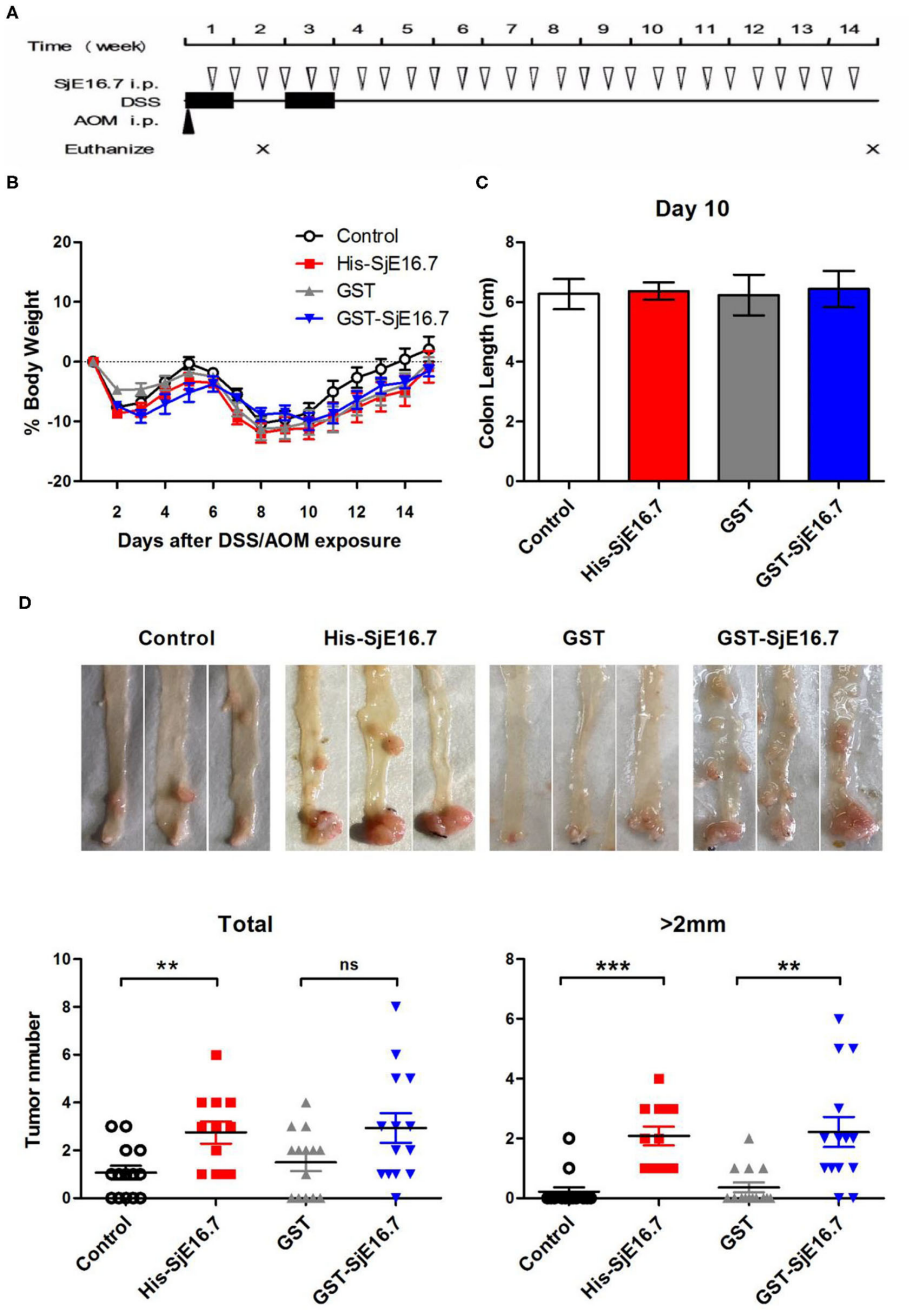
SjE16.7 promotes colon-rectal cancer development in mice. **(A)** Illustration of the experimental layout of CAC induction and *in vivo* treatment with recombinant protein His-SjE16.7, GST-SjE16.7, and GST control. **(B)** Change in body weight over time is expressed as the percentage of the initial body weight. Pooled data from four independent experiments is shown. *n* = 15–20 per group. **(C)** Colon length was measured on day 10, *n* = 5 per group. **(D)** Colonic neoplasms were photographed and numbers of macroscopic neoplasms were counted individually at week 14. Results are presented as pooled data from three independent experiments. **p < 0.01, ***p < 0.001 compared with the indicated control group.

**Figure 6:**
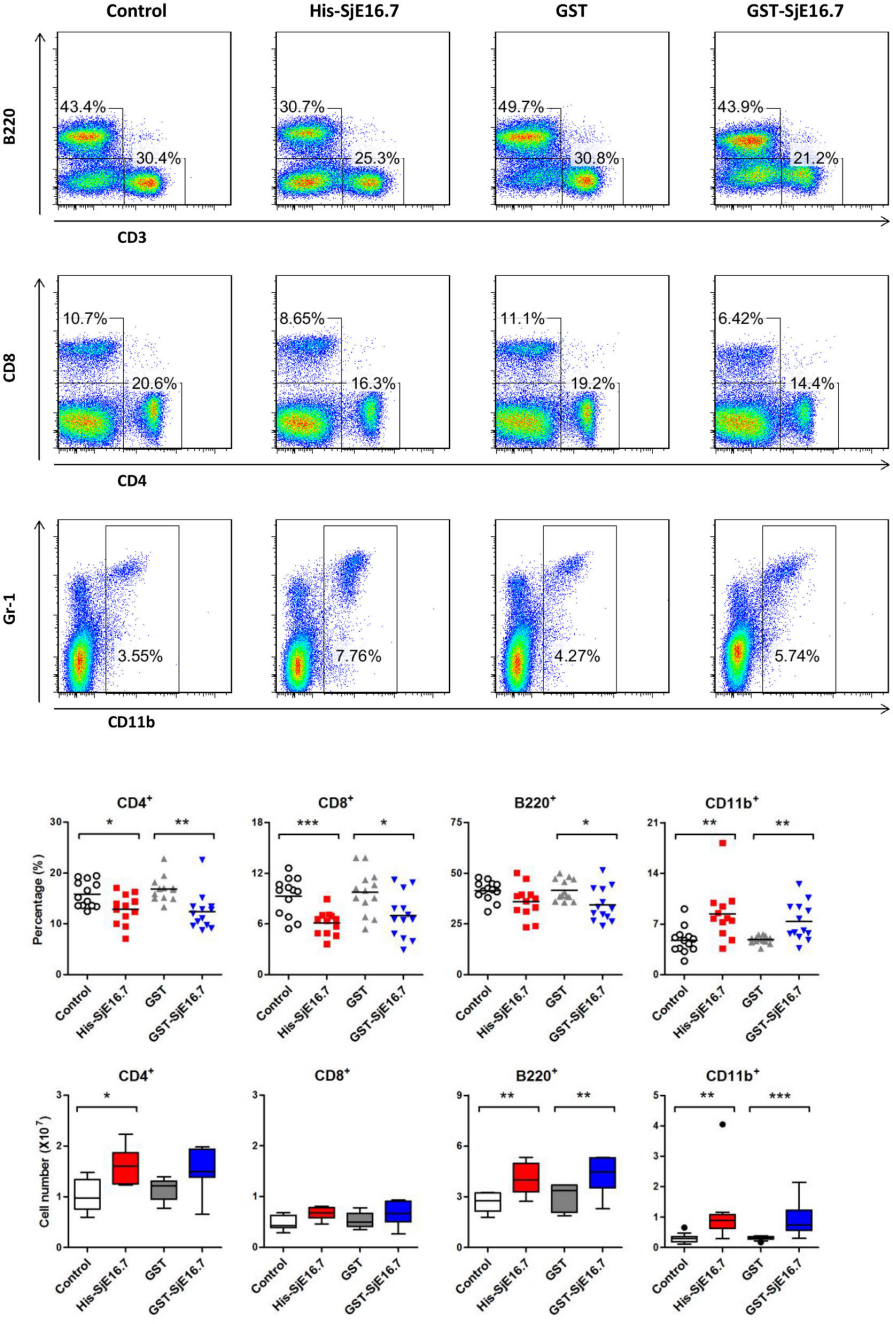
SjE16.7 administration increases the frequency of myeloid cells in the spleen of the CAC mouse model. Splenocytes were isolated from His-SjE16.7, GST-SjE16.7, GST-control, or PBS control-treated mice 14 weeks post-CAC induction. Frequencies of splenic B220^+^, CD3^+^, CD4^+^, CD8^+^, and CD11b^+^ cells were determined by flow cytometry. The frequency and number of cells in spleen is presented as the mean ± SEM of 12–13 individual mice per group. *p < 0.05, **p < 0.01, ***p < 0.001 compared with the indicated control group.

**Figure 7:**
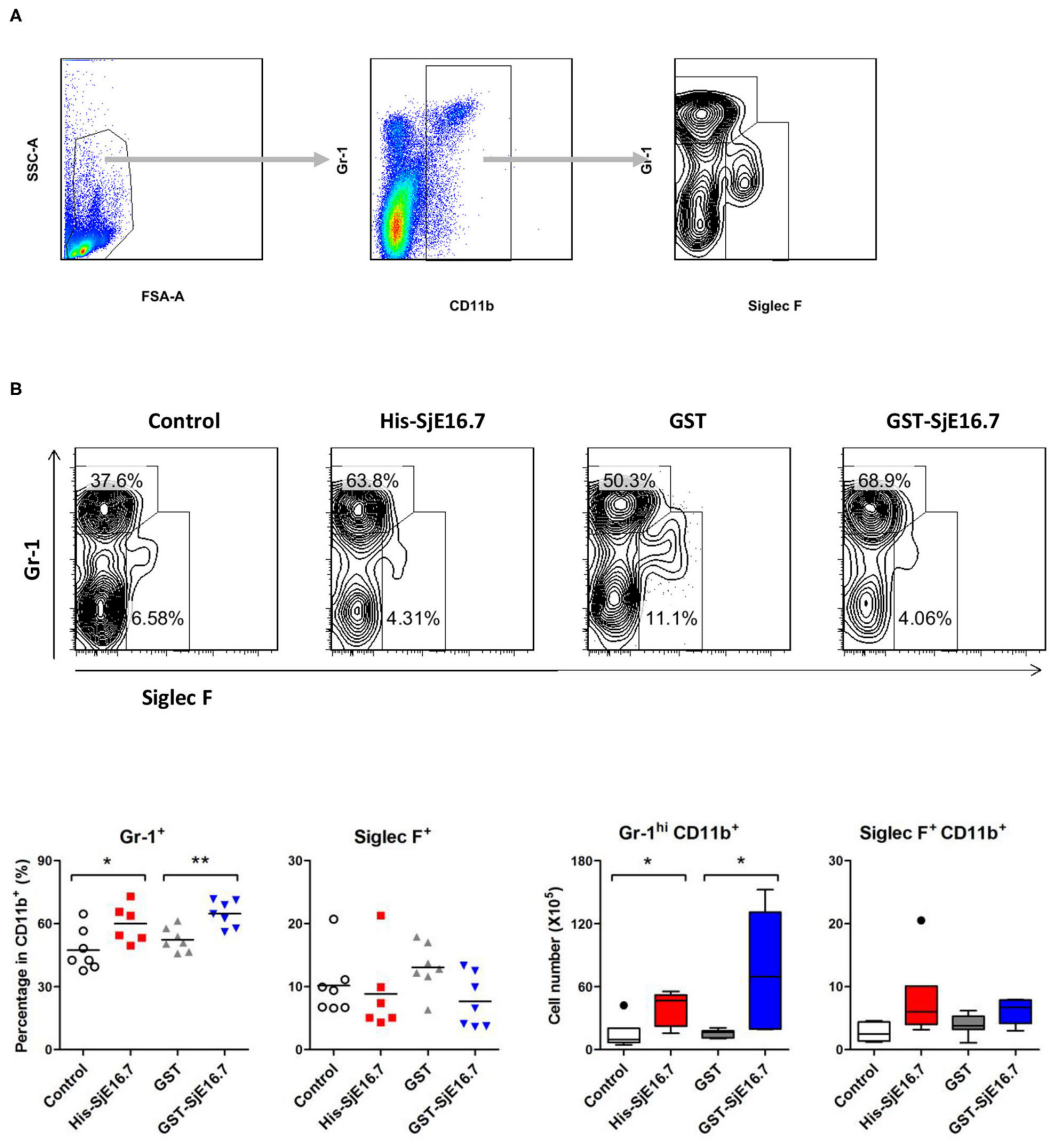
SjE16.7 administration increases CD11b^+^Gr-1^hi^ myeloid cells in the spleen of the CAC mouse model. Splenocytes were isolated from His-SjE16.7, GST-SjE16.7, GST-control, or PBS control treated mice 14 weeks post-CAC induction. **(A)** Representation of the flow cytometry gating strategy for the determination of splenic CD11b^+^ myeloid cells subpopulations. **(B)** Frequency and absolute number of Gr-1^hi^ and Siglec F^+^ cells within the CD11b^+^ myeloid population was determined by flow cytometry. The frequency and number of CD11b^+^Gr-1^hi^ and CD11b^+^Siglec F^+^ cells in spleen are presented as the mean ± SEM of six to seven individual mice per group. *p < 0.05, **p < 0.01 compared with indicated control group.

**Figure 8:**
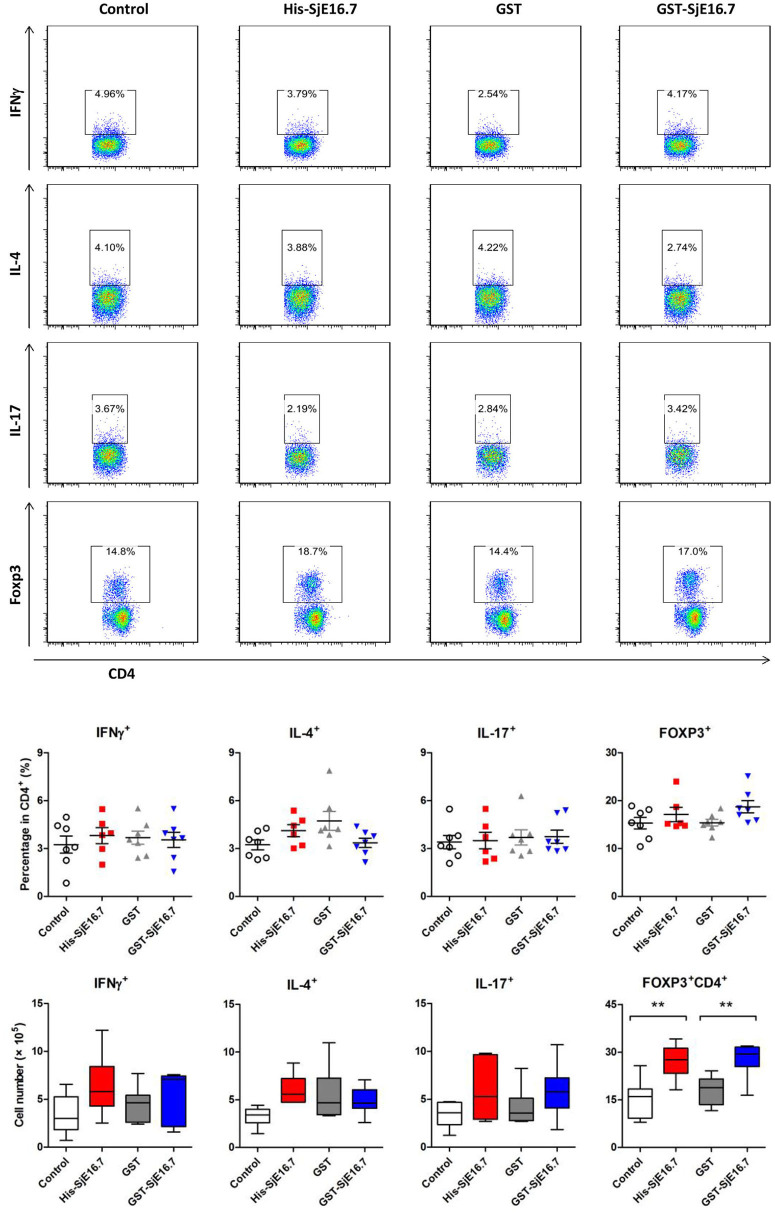
SjE16.7 administration increases splenic FoxP3+ CD4^+^ T cell numbers in CAC mice treated with or without SjE16.7. Splenocytes were isolated from His-SjE16.7, GST-SjE16.7, GST-control, or PBS control treated mice 14 weeks post-CAC induction, and intracellular staining was performed as described in Materials and Methods. The frequencies of IFN-γ^+^, IL-4^+^, IL-17^+^, and Foxp3^+^ cells within the CD4^+^ population were determined by flow cytometry. The frequency and absolute number of CD4^+^ IFN-γ^+^, CD4^+^IL-4^+^, CD4^+^IL-17^+^, and CD4^+^Foxp3^+^ cells in spleens are presented as the mean ± SEM of six to seven individual mice per group. **p < 0.01, compared with indicated control group.

In order to investigate if SjE16.7 contributes to inflammation-induced tumor development, we analyzed the role of SjE16.7 in a mouse model of colitis-associated colorectal cancer (CAC). CAC was induced in mice using a single dose of AOM followed by repeated cycles of DSS. Separate groups of AOM-DSS mice were treated with vehicle control (PBS), control protein (GST), GST-SjE16.7, or His-SjE16.7 (Figure 5A). Animals in all groups exhibited weight loss and diarrhea during the acute phase, which resolved within 2 weeks after AOM/DSS treatment (Figure 5B). Mice treated with eukaryotic His-SjE16.7 and prokaryotic GST-SjE16.7 did not show any significant difference in weight loss or clinical signs of inflammation compared to the controls (Figures 5B,C). However, 14 weeks after initiation, the incidence of colorectal tumors was increased in SjE16.7-treated animals with 100% incidence in the His-SjE16.7 treated group and 90% incidence in the GST-SjE16.7 treated group although the tumor incidences in the PBS group and GST control group were 50 and 70%, respectively. SjE16.7 treatment resulted in significantly more large tumors (>2 mm) than in the control groups (Figure 5D). As expected from their increased tumor numbers and tumor sizes, His-SjE16.7- and GST-SjE16.7-treated mice had a significant increase in the frequency of inflammatory myeloid cells (CD11b^+^) in their spleens but significantly lower frequency of CD4^+^ and CD8^+^ T cells (Figure 6). Further analysis of myeloid and T-cell subsets showed that the frequency and numbers of Gr-1^hi^ myeloid cells was significantly higher in the His-SjE16.7 and GST-SjE16.7 treated mice, and the frequency and numbers of SiglecF^+^ eosinophils, IFN-γ^+^, IL-4^+^, and IL-17^+^ CD4^+^ T helper cells were similar between the antigen-treated and control groups (Figures 7, 8). The number of FoxP3^+^CD4^+^ T regulatory cells, however, was significantly increased in the His-SjE16.7 and GST-SjE16.7 treated mice, compared to controls (Figure 8).

Taken together, the data demonstrate that SjE16.7 exposure leads to increased colorectal tumor development and is associated with a higher frequency of myeloid inflammatory cells in the spleen.

## Discussion

Several parasitic infections are associated with carcinogenesis and human malignancy (4). The helminth diseases opisthorchiasis and clonorchiasis are strongly associated with cholangiocarcinoma, and schistosomiasis is associated with bladder carcinoma and colorectal cancer, depending on the schistosome species. However, some parasites may also act as negative regulators of cancer. This includes the activation of NK cells and CD8^+^ T cells seen during *Toxoplasma gondii* infection and the pro-apoptotic effects of *Trypanosoma cruzi* infection as well as the Kunitz type protease inhibitor from *Echinococcus granulosus*, which was shown to induce tumor cell apoptosis in a mouse melanoma model (14, 15).

Although infection with *S. japonicum* is a known risk factor for the development of colorectal cancer, the exact etiopathogenesis of schistosome-associated colorectal cancer remain enigmatic (16–18). Here, we provide evidence that the schistosome SjE16.7 protein may contribute to colorectal cancer progression. This conclusion is based on the following observations: (a) SjE16.7 binds to RAGE, which is known to have a key role in inflammation-associated cancer development. (b) SjE16.7-RAGE ligation leads to an increase in ROS generation, NF-κB signaling activation, and pro-inflammatory cytokine production, which all contribute to establishing a protumorigenic environment. (c) *In vivo* mouse experiments demonstrate that intraperitoneal injection of SjE16.7 promote colitis-associated colorectal cancer progression along with systemic myeloid cell accumulation.

RAGE is a pattern recognition receptor that can bind multiple ligands (19). Among these ligands, the S100 proteins are small calcium-binding proteins with EF-hand motifs prevalent in a variety of inflammatory diseases and known contributors to cancer cell biology (10). Similar to S100 proteins, SjE16.7 is a small calcium-binding protein with 2 EF-hand motifs. We have previously demonstrated that SjE16.7 is a potent myeloid cell activator used by the schistosome egg to mediate the inflammatory response needed to facilitate the passage of eggs through tissues (6, 7). In this study, we have established that SjE16.7 binds to RAGE, leading to activation of downstream signaling pathways. There is evidence that the S100 proteins form subgroups that bind to different sites on RAGE, resulting in triggering of distinct cellular pathways (11). The binding domain of the receptor or the biding epitope on SjE16.7 is currently unknown, and future studies are necessary to increase our detailed understanding of the interaction between SjE16.7 and mammalian cells.

RAGE is expressed on many different cell types (13, 20–23). Considering that SjE16.7 is a potent innate immune cell activator and that chronic inflammatory processes are essential in schistosomiasis-associated cancer (17, 24), we focused on SjE16.7-induced RAGE signaling in innate immune cells. We found that SjE16.7-RAGE ligation on the macrophage surface triggers the generation of ROS and the release of pro-inflammatory cytokines (IL-6 and TNF-α) and that the NF-κB pathway is involved in the RAGE-dependent macrophage activation. Thus, our results are consistent with previous reports suggesting that RAGE is a central mediator of innate immune responses (20). Our study also implies that SjE16.7 may be involved in schistosomiasis-associated cancer progression via RAGE ligation. RAGE signaling is known to drive the strength and maintenance of an inflammatory response and, as such, serve as a key player in bridging chronic inflammation and cancer (25), and using the murine CAC model, we found that *in vivo* treatment with SjE16.7 does indeed promote colorectal tumor progression. The development of colon cancer is a multistep process that is regulated by various intrinsic and extrinsic cellular signals (26, 27), and activation of RAGE signaling in innate immune cells by SjE16.7 may result in the establishment of a protumorigenic microenvironment through the secretion of pro-inflammatory cytokines such as IL-6, and TNF-α. It should be noted, however, that there is also evidence that IL-6 and TNF-a may be antitumorigenic at higher concentrations, suggesting that the local concentration of these cytokines in the tumor microenvironment is a key factor in tumor progression (28–32).

Our CAC model experiments demonstrated that SjE16.7-treated mice had increased numbers of tumors and increased size of tumors along with an increased frequency of CD11b^+^ myeloid cells in the spleen. The increase in myeloid cell frequencies was accompanied by a decrease in T cell frequencies for both CD4^+^ and CD8^+^ T cells. Accumulation of myeloid-derived suppressor cells (MDSCs) is one of the major immunological abnormalities in cancer resulting in T cell tolerance and suppression of antitumor immune response. MDSCs are CD11b^+^Gr1^+^ cells (CD11b^+^Ly6G^+^ or CD11b^+^Ly6C^+^) with the ability to prevent T cell activation, and they have been identified in the spleen, blood, and tumor tissues of tumor-bearing mice (21, 33, 34). Importantly, it has been reported that RAGE signaling contributes to the development of MDSCs, and that RAGE stimulation of MDSCs promote their migration and accumulation through NF-κB signaling pathways (35, 36). In our study, SjE16.7 treated mice had higher frequencies and cell numbers of CD11b^+^Gr-1^hi^ myeloid cells compared to the control groups. This phenotype suggests that SjE16.7 may promote MDSC development and/or accumulation via RAGE ligation. The frequencies and absolute numbers of splenic Th1, Th2, and Th17 cells were no different between groups treated with or without SjE16.7 in our CAC model. The absolute number of splenic Treg cells, however, was significantly increased in the SjE16.7-treated groups. It has previously been reported that MDSC promotes the induction of Tregs and that the two cell types may collaborate in suppressing antitumor immunity (37). Further experiments combining *in vitro* and cell function assays are necessary to establish whether this is the case. Moreover, it is important to establish whether this is a specific mechanism for colorectal cancer or it could also be similar in other types of cancer, such as schistosomiasis-associated liver cancer. In addition to immune response, multiple other factors, such as oncogene activation, inhibition of tumor suppressors, modifications of normal microRNA expression patterns, alterations to the epigenetic landscape as well as the intestinal microbiota are also involved in colorectal cancer development (38). RAGE is present on epithelial and mesenchymal cells and is known to play a role in the development of sporadic intestinal tumors (26). Our *in vitro* data demonstrate that SjE16.7 binds to RAGE on epithelial cells, however, whether SjE16.7 also activates RAGE signaling pathways in epithelial cells, similar to that seen in myeloid cells, and if so, whether this contribute to its protumorigenic function *in vivo* still needs to be determined.

A number of mouse models are available for the study of colon carcinogenesis (39), including transgenic and chemically induced models. The latter group include *in vivo* treatments with chemical agents, such as methylnitrosourea (MNU), 2-amino-1-methyl-6-phenylimidazol [4,5-b]pyridine (PhIP), or 1,2-dimethylhydrazine (DMH) (40). However, among the chemically induced mouse tumor models, the combination of a single dose of AOM followed by 1 week exposure to the inflammatory agent DSS has proven to dramatically shorten the time for tumor development. Because of this, together with its high reproducibility and potency as well as the simple and affordable mode of application, the AOM/DSS has become a superior model for studying colon carcinogenesis (39). The AOM/DSS model is particularly useful when the focus is on tumor progression driven by colitis as an inflammatory disease (41). In this study, we used the AOM/DSS model to demonstrate that SjE16.7 contributes to this form of inflammation-induced cancer. Future studies will be aimed at establishing if SjE16.7 also acts as an oncogenic driver in other types of cancer models.

In addition to the association of schistosomiasis infection and colorectal cancer, infections with the urogenitary species, *S. haematobium*, has been correlated with the occurrence of bladder carcinoma (42). According to the genome information of schistosomes, there are homologs of SjE16.7 in both *S. haematobium* (70% identity) and *S. mansoni* (SmE16, 70% identity) (43, 44). It has been reported that SmE16 is one of the more abundant *S. mansoni* egg secreted proteins; however, there is currently limited information available regarding the specific roles of the *S. mansoni* and *S. haematobium* homologs of SjE16.7, and more research in this field is needed.

In conclusion, our study demonstrates a key role for the schistosome egg protein SjE16.7 in driving pro-inflammatory innate responses by acting as a ligand for RAGE. In addition, our data provide additional insight into the role played by SjE16.7 in promoting tumor progression.

## Data Availability Statement

The datasets presented in this study can be found in online repositories. The names of the repository/repositories and accession number(s) can be found in the article.

## Ethics Statement

The animal study was reviewed and approved by the Animal Ethics Committee of Shanghai Jiao Tong University School of Medicine.

## Author's Note

Infections may promote cancer development because infectious agents induce procarcinogenic inflammatory processes within their host. Schistosomiasis, caused by infection with blood fluke trematodes of the genus Schistosoma, is one of the most important human parasitic diseases across the world. Schistosomiasis is associated with urinary bladder cancer, colorectal cancer, rectal cancer, and hepatocellular carcinoma, but the underlying mechanisms are still not well-understood. SjE16.7 is a calcium binding protein secreted from *Schistosoma japonicum* eggs. We have previously demonstrated that SjE16.7 is a neutrophil attractant and macrophage activator and initiates the inflammatory granuloma responses in schistosomiasis. In this study, we report that SjE16.7 binds to host cells by interacting with receptors for advanced glycation end products (RAGE). This ligation leads to an increase in the generation of reactive oxygen species and production of pro-inflammatory cytokines. *In vivo* experiments demonstrate that SjE16.7 treatment promotes colitis-associated colorectal cancer progression along with systemic myeloid cell accumulation. Our data suggest that SjE16.7 is associated with colorectal cancer due to the activation of procarcinogenic inflammatory processes. Thus, our results identify a new helminth antigen contributing to tumor development in the mammalian host.

## Author Contributions

ZW and GC conceived of the study. CW, XD, ZW, and HH wrote the article. CW, XD, and LT designed and performed the experiments and analyzed the data. XD, JW, WZ, XG, DL, and WH were involved the design or execution of several experiments. ZW supervised the study. All authors contributed to the article and approved the submitted version.

## Conflict of Interest

The authors declare that the research was conducted in the absence of any commercial or financial relationships that could be construed as a potential conflict of interest.
